# Community Pattern of Ectoparasite and Endoparasite Fauna of Urban‐Dwelling Anurans in the Southwestern Coast of Sarawak, Borneo Island, Malaysia

**DOI:** 10.1155/japr/6409751

**Published:** 2026-09-23

**Authors:** Dayangku Batrisyia Qistina Aneez Awang Raduan, Zalifah Husna Ali, Abang Haziq Abang Ismail, Ahmad Shahmi Abdul Salim, Ramlah Zainudin, Madinah Adrus

**Affiliations:** ^1^ Faculty of Resources Science and Technology, Universiti Malaysia Sarawak, Kota Samarahan, Sarawak, Malaysia, unimas.my

**Keywords:** anuran, parasite diversity, prevalence, urban, urbanization

## Abstract

**Background:**

Urbanization has greatly influenced ecological interactions, impacting both host species and their parasites. Gaining insight into the relationship between parasites and urban anurans is vital for enhancing public health awareness. This study documented parasite fauna in anurans across urban areas in Kuching and Kota Samarahan, Sarawak, Borneo Island.

**Results:**

A total of 170 individuals of urban anurans from five families and eight species were examined for ectoparasites and endoparasites. Across 170 urban anurans, infection prevalence reached 51.2% (*n* = 87). *Duttaphrynus melanostictus* exhibited the highest parasite burden and diversity, particularly for gastrointestinal (47.8% of *D. melanostictus* individuals) and blood parasites (10.0%), and showed frequent coinfections. Ectoparasites were infrequent and restricted to this host. Seven out of 10 parasite species are of potential zoonotic concern.

**Conclusion:**

These results offer a baseline overview of parasite fauna in urban anurans, identifying rainfall and temperature as key environmental drivers of infection and community composition and highlighting zoonotic risks that warrant ongoing public surveillance.

## 1. Background

In the fast‐paced environment of contemporary society, the race to construct towering skyscrapers and extensive residential and industrial complexes intensifies to accommodate the demands of a growing global population [1]. This urban expansion results in increased waste production, as the density of human settlements rises annually [2]. In the absence of effective waste management strategies, the risk of disease outbreaks escalates [3]. Anurans, which have evolved to thrive in urban settings, are also affected by the detrimental impacts of urbanization. Polluted environments disrupt the immune systems of these amphibians, rendering them more vulnerable to diseases and parasitic infections, which pose significant health risks [4].

The rapid pace of urbanization exerts profound effects on the environment, particularly concerning amphibians. Their unique lifestyle, which spans both land and water, makes them particularly susceptible to adverse environmental changes [5]. The relationship between animal parasites and their hosts is also influenced by urbanization. Changes in the physicochemical environment can have direct repercussions for parasites [6], impacting the physiology, behavior, and overall fitness of their hosts. As noted by [7], urban settings can facilitate the transmission and proliferation of animal parasites and pathogens.

In Malaysia, particularly in Sarawak, located in the southwestern region of Borneo Island, research on urban parasites affecting anurans remains sparse, with most investigations concentrated on Peninsular Malaysia [8]. Studies by [9] explored the parasitic species found in two wild anuran species from a riverine habitat in Penang, examining how various environmental factors influence anuran populations and their associated parasites. Other research, such as by [10], focused on protozoan parasites in anurans at Zoo Negara, Malaysia, whereas [11] studied intestinal parasites in various zoo animals in Malaysia. Some nematodes identified in anuran intestines have also been documented [9]. These nematode infections are mainly indicative of soil‐transmitted helminth (STH) infections, which typically arise from direct soil contact.

Investigating the relationships between parasites and their hosts in urban environments is crucial, especially given the emergence of wildlife diseases linked to human‐induced environmental changes. Nonetheless, the underlying mechanisms of these patterns remain largely unexplored [12]. Changes in the distribution of hosts and vectors may play a role in the emergence of diseases [12, 13]. Therefore, this study is aimed at characterizing the community patterns of ecto‐ and endoparasites associated with urban‐dwelling anurans located on the southwestern coast of Sarawak, Malaysian Borneo, and evaluating the influence of host identity, urbanization intensity, and environmental variables on infection prevalence and parasite diversity. The outcomes will aid in formulating a conservation strategy focused on sustaining and enhancing the diversity of anuran populations.

## 2. Methods

### 2.1. Study Area

**Figure 1:**
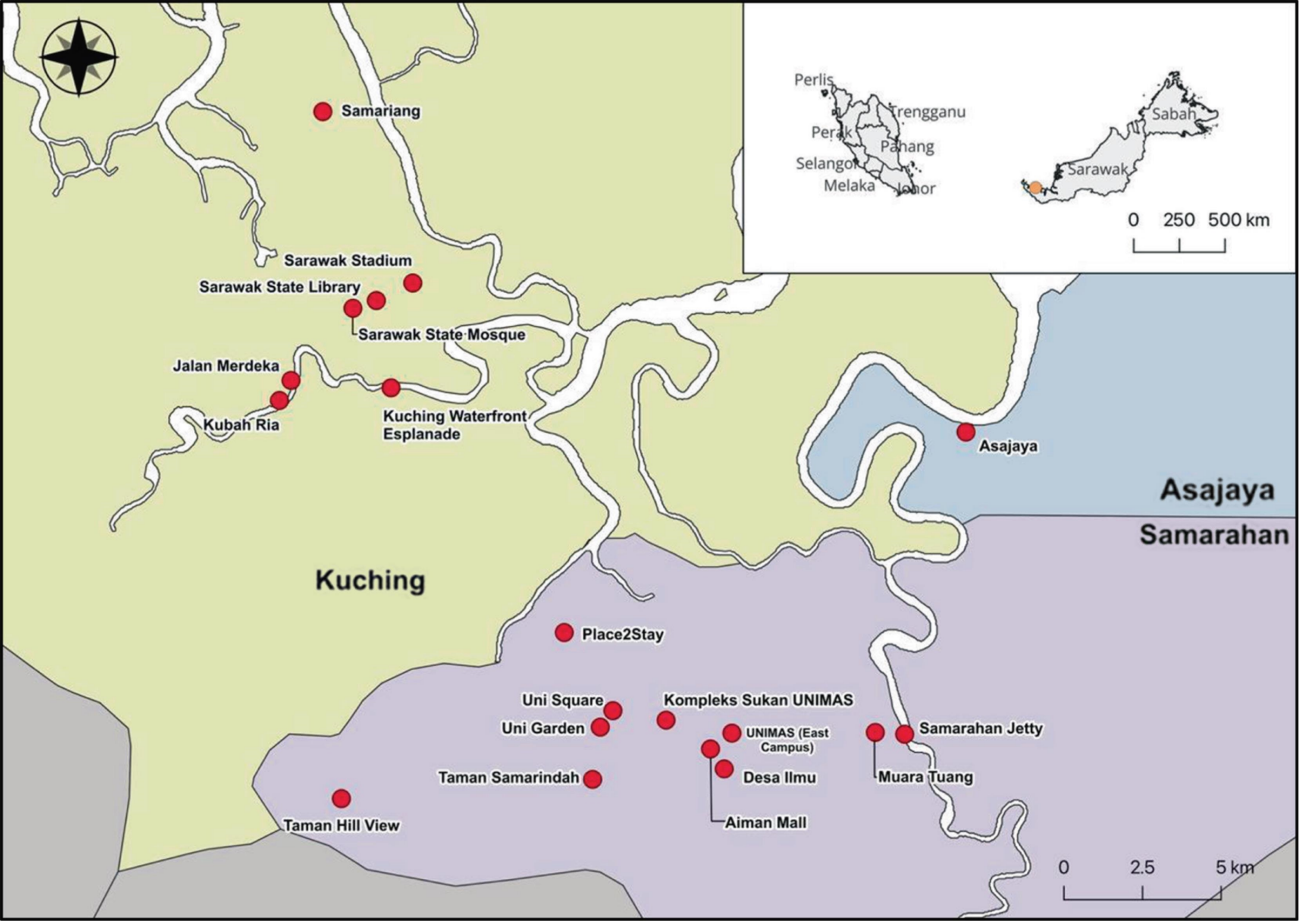
Study areas of 12 localities in Kota Samarahan and six localities in Kuching.

Nineteen sampling localities were selected across two administrative jurisdictions in southwestern Sarawak, the Commission of the City of Kuching North (six sites) and Kota Samarahan Municipal Council (13 sites) (Figure 1; Table 1). Sites were classified into three urbanization disturbance categories, low (*n* = 4), medium (*n* = 10), and high (*n* = 5) based on land use type and degree of habitat modification [4, 13, 14]. Low disturbance sites comprised seminatural areas with minimal infrastructure. Medium disturbance sites included residential and sports facilities with moderate human activity. High disturbance sites were heavily urbanized commercial and city center areas with dense infrastructure and elevated waste accumulation.

**Table 1:** Coordinate of each locality and their descriptions.

Location	Area	Coordinate	Description
Commission of The City of Kuching North	● Sarawak State Library (PUSTAKA)	1° 35 ^′^N, 110° 21 ^′^E	Recreational park
	● Samariang	1° 38 ^′^N, 110° 20 ^′^E	Housing area
	● Sarawak State Mosque	1° 33 ^′^N, 110° 20 ^′^E	City center
	● Sarawak Stadium	1° 35 ^′^N, 110° 21 ^′^E	Sports center
	● Jalan Merdeka	1° 34 ^′^N, 110° 20 ^′^E	Village road
	● Kubah Ria	1° 33 ^′^N, 110° 20 ^′^E	Fresh market
	● Kuching Waterfront esplanade	1° 34 ^′^N, 110° 21 ^′^E	City center

Kota Samarahan Municipal Council (MPKS)	● Kampus Timur UNIMAS	1° 28 ^′^N, 110° 26 ^′^E	University
	● Kompleks Sukan UNIMAS	1**°** 45 ^′^N, 110° 26 ^′^E	University
	● Desa Ilmu	1° 27 ^′^N, 110° 27 ^′^E	Housing area
	● Uni Garden	1° 27 ^′^N, 110° 27 ^′^E	Housing area
	● Samarahan jetty	1° 28 ^′^N, 110° 25 ^′^E	Jetty
	● Taman Samarindah	1° 40 ^′^N, 110° 24 ^′^E	Housing area
	● Uni Square	1° 27 ^′^N, 110° 25 ^′^E	Housing area
	● Place 2 Stay	1° 29 ^′^N, 110° 24 ^′^E	Hotel
	● Taman Hill View	1° 26 ^′^N, 110° 20 ^′^E	Housing area
	● Muara Tuang	1° 27 ^′^N, 110° 29 ^′^E	Housing area
	● Asajaya	1° 29 ^′^N, 110° 29 ^′^E	Housing area
	● Aiman Mall	1° 27 ^′^N, 110° 26 ^′^E	Business center

At each site, ambient temperature (°C), relative humidity (%), rainfall (mm/day), and water or soil pH were recorded per sampling session. Temperature and humidity were measured using a digital thermohygrometer, pH was measured in situ with a calibrated portable meter, and rainfall data were extracted from the NASA POWER database using site coordinates.

### 2.2. Host Collection

Anurans were collected nocturnally (19:00–22:00 h) using the visual encounter survey (VES) method, following peak anuran activity. Each individual was housed separately to prevent cross‐contamination, labeled with sampling number, location, date, time, activity state, and distance from the nearest water source, and transported to the laboratory for identification and parasitological examination. Species identification followed published Bornean amphibian field guides [15, 16]. Snout–vent length, tibia length, and body mass were recorded for each individual.

### 2.3. Parasitological Analysis

All procedures complied with Universiti Malaysia Sarawak Animal Ethics Committee (UNIMAS/AEC/R/F07/046). Permission to conduct a biological resource survey was granted by the Controller of Wildlife, Forest Department Sarawak (Permit No. [137] JHS/NCCD/600‐7/2/Jld.2). Anurans were euthanized using MS‐222 Tricaine methanesulfonate prior to dissection. All parasitological procedures followed standard methods as described below.

### 2.4. Ectoparasite

Ectoparasites were examined by observing the whole morphology of the host under the stereoscopic microscope. Arthropod ectoparasites such as ticks and mites were collected using forceps and kept in a vial filled with a 70% ethanol solution. Ectoparasites were collected and cleared by submerging them in lactophenol [17, 18]. The abdomen was punctured laterally and mounted using modified Canada Balsam solution mixed with xylene. Species were identified using available keys, published taxonomic drawings, and references [19, 20].

### 2.5. Endoparasite

#### 2.5.1. Gastrointestinal Parasites

The host body was dissected using a sterile scalpel, and the stomach, intestines, rectum, heart, and liver were removed and examined individually. Gastrointestinal parasites were collected using fine‐tipped forceps, transferred into vials, and preserved in 75% ethanol for mounting and identification. Fecal samples were examined using both flotation and sedimentation techniques [21]. Flotation solution was prepared by dissolving 420 g of sodium chloride into 1000 mL of distilled water under medium heat until dissolved [22]. Fecal material was mixed with flotation solution and centrifuged for 10 min, and recovered eggs were mounted on glass slides in glycerol and methyl blue and then examined at magnifications of 40×, 100×, and 1000×. Parasite identification was based on published reference keys [21, 23].

#### 2.5.2. Blood Parasites

Blood was collected from the ventral abdominal vein or the ventricle of the heart [24]. Samples were collected by cardiac puncture using a 1‐mL syringe. Both thin and thick blood films were prepared. For thin films, a small drop of blood was spread at a 45° angle on a clean slide, then air‐dried, methanol‐fixed, and stained with Giemsa. For the thick blood smear, a few drops of blood were spread in a circular motion, air‐dried for 30 min, and submerged in Giemsa before examination at varying magnifications (400×–1000×). Any observed parasites were identified based on Davies and Johnston′s [23] and Wahab et al.′s [9] work.

### 2.6. Data Analysis

All analyses were conducted in R v4.5.2 [25] using *tidyverse*, *vegan*, and *FSA* packages (*α* = 0.05). Infection prevalence (%) was calculated per Bush et al. [26]. Chi‐square tests compared prevalence among host species (with caution when expected counts < 5). Relationships between environmental variables (rainfall, temperature, humidity, and pH) and parasite metrics (prevalence, Shannon diversity *H* ^′^, and mean richness) were assessed using Spearman′s correlation. The Kruskal–Wallis tests with Dunn′s tests with Bonferroni correction compared parasite metrics across disturbance categories. A binomial generalized linear model (GLM) with a logit link and AIC‐based backward selection modeled individual infection status. Community composition was analyzed using NMDS (Bray–Curtis), with *envfit* and PERMANOVA (999 permutations) for group‐level differences.

## 3. Results

**Figure 2:**
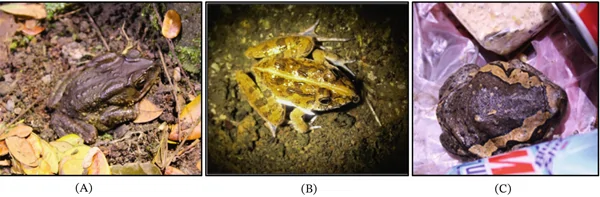
(A) *Duttaphrynus melanostictus* is the most common anuran host found in urban areas, followed by (B) *Fejervarya limnocharis* and (C) *Kaloula pulchra.*

A total of 170 anurans belonging to eight species from five families were collected across 19 urban localities in southwestern Sarawak (Table 2). The most abundantly sampled species were *Duttaphrynus melanostictus* (Bufonidae; *n* = 90), *Fejervarya limnocharis* (Dicroglossidae; *n* = 29), *Kaloula pulchra* (Microhylidae; *n* = 25) (Figure 2), and *Chalcorana raniceps* (Ranidae; *n* = 20). The remaining four species, *Haplobatrachus rugulosus*, *Hylarana baramica*, *Limnonectes paramacrodon*, and *Polypedates leucomystax*, were each represented by three or fewer individuals and were, therefore, excluded from statistical comparisons because prevalence estimates derived from *n* ≤ 3 are statistically unstable. These values are reported solely for completeness and should not be interpreted as reliable estimates.

**Table 2:** Prevalence of parasitic infections in urban anurans from the southwestern coast of Borneo Island, Sarawak.

Anuran species	Total no. examined	Total no. of infected	No. of hosts (Anura) infected		
			Ectoparasites	Endoparasites	
				Blood parasites	Gastrointestinal parasites
Family: Bufonidae					
*Duttaphrynus melanostictus*	90	54 (60.0%)	4 (4.4%)	9 (10.0%)	43 (47.8%)
Family: Dicroglossidae					
*Fejervarya limnocharis*	29	8 (27.6%)	0	4 (13.8%)	5 (17.2%)
*Limnonectes paramacrodon* ^a^	1	0	0	0	0
*Haplobatrachus rugulosus* ^a^	3	0	0	0	0
Family: Microhylidae					
*Kaloula pulchra*	25	18 (72.0%)	0	4 (16.0%)	14 (56.0%)
Family: Ranidae					
*Chalcorana raniceps*	20	6 (30.0%)	0	6 (30.0%)	0
*Hylarana baramica* ^a^	1	1 (100%)^a^	0	0	1 (100%)^a^
Family: Rhacophoridae					
*Polypedates leucomystax* ^a^	1	0	0	0	0

Total	170	87 (51.2%)	4 (2.4%)	23 (13.5%)	63 (37.1%)

*Note:* Prevalence (%) is calculated as the number of infected hosts ÷ total number examined × 100. The sum of infected hosts across parasite categories may exceed the total number of infected individuals due to coinfections, whereby a single host was simultaneously infected with parasites from more than one category.

^a^Prevalence estimates for species with *n* ≤ 3 should be interpreted with caution due to small sample size.

Overall infection prevalence was 51.2% (*n* = 87) (Table 2). Ten parasite taxa were recorded (one ectoparasite, three blood parasites, and six gastrointestinal parasites) (Table 3). The sole ectoparasite, *Scheloribates* sp. (Oribatida), was found exclusively on *Duttaphrynus melanostictus.* Among blood parasites, *Hepatozoon* spp. was the most prevalent, infecting three host species, followed by *Trypanosoma* spp. (*n* = 4) and *Lankesterella* spp. (*n* = 2). Gastrointestinal parasites were the most abundant and diverse group, with *Macracanthorhynchus* spp. and *Strongyloides* spp. recording the highest prevalence. *Duttaphrynus melanostictus* harbored all 10 taxa and showed the most frequent coinfections. Host species identity significantly influences infection status (*χ* ^2^ = 16.87, df = 7, *p* = 0.018).

**Table 3:** Parasite species recorded and number of hosts infected in urban anurans from the southwestern coast of Borneo Island, Sarawak.

Parasites species	Urban anuran species infected								Total no. of individuals	Number of host species infected	Prevalence infected (%)
	*Fejervarya limnocharis*	*Hoplobatrachus rugulosus*	*Duttaphrynus melanostictus*	*Limnonectes paramacrodon*	*Chalcorana raniceps*	*Hylarana baramica*	*Kaloula pulchra*	*Polypedates leucomystax*			
Ectoparasites	—	—	4	—	—	—	—	—	4	1	2.4
*Scheloribates* spp.											
Endoparasites											
Blood parasites											
*Hepatozoon* spp.	—	—	3	—	5	—	2	—	10	3	5.9
*Lankesterella* spp.	2	—	4	—	—	—	—	—	6	2	3.5
*Trypanosoma* spp.^a^	2	—	2	—	1	—	2	—	7	4	4.1
Gastrointestinal parasites											
*Heterakis* spp.	1	—	8	—	—	1	3	—	13	4	7.6
*Macracanthorhynchus* spp.^a^	3	—	21	—	—	—	—	—	24	2	14.1
*Strongyloides* spp.^a^	—	—	16	—	—	—	9	—	25	2	14.7
*Entamoeba* spp.^a^	1	—	13	—	—	—	7	—	21	3	12.4
*Ascaris* spp.^a^	1	—	5	—	—	1	5	—	12	4	7.1
*Trichuris* spp.^a^	—	—	4	—	—	—	—	—	4	1	2.4

Number of parasite species recorded	6	—	10	—	2	2	6	—			
Total number of Anuran examined	29	3	90	1	20	1	25	1			
Total number of anurans infected	8	0	54	0	6	1	18	0			

*Note:* “—” indicates no infection detected. Prevalence (*%*) = number of hosts infected ÷ total anurans examined (*n* = 170) × 100.

^a^Potential zoonotic concern species.

Infection prevalence increased progressively from low to high disturbance sites, though the Kruskal–Wallis test detected no statistically significant difference across disturbance categories for any parasite metric (all *p* > 0.05; Tables 4 and 5). The absence of significant differences may partly reflect limited statistical power arising from the small and uneven number of sampling sites among disturbance categories (low: *n* = 4; medium: *n* = 10; and high: *n* = 5). Sensitivity analysis indicated that this sampling design was capable of detecting only large effects (Cohen′s *f* = 0.784 at 80% power and *α* = 0.05), suggesting that smaller ecological differences may have remained undetected.

**Table 4:** Infection prevalence and parasite diversity across urbanization disturbance categories.

Disturbance level	No. of sites	No. of anurans	Infected	Prevalence (%)	Shannon diversity (*H* ^′^)
Low	4	26	10	38.5	0.84 ± 0.57
Medium	10	85	37	43.5	0.37 ± 0.42
High	5	34	19	55.9	0.76 ± 0.59

**Table 5:** The Kruskal–Wallis test results comparing parasite metrics across urbanization disturbance categories.

Parasite metric	*H* statistic	*d* *f*	*p* value	Significant
Infection prevalence	1.117	2	0.572	No
Shannon diversity (*H* ^′^)	2.879	2	0.237	No
Mean parasite richness	1.249	2	0.535	No

Abbreviation: ns, not significant (*p* > 0.05).

Rainfall was the most consistent environmental predictor at the site level, with a significant positive association with infection prevalence (*ρ* = 0.607, *p* = 0.006) and mean parasite richness (*ρ* = 0.574, *p* = 0.010). Temperature was significantly and positively associated with both infection prevalence (*ρ* = 0.520, *p* = 0.022) and mean richness (*ρ* = 0.509, *p* = 0.026). In contrast, neither pH nor humidity showed significant correlations with any parasite metric (all *p* > 0.05) (Table 6).

**Table 6:** Spearman′s rank correlations between environmental variables and parasite metrics across 19 sampling sites.

Environmental variable	Parasite metric	*ρ* (rho)	*p* value	Significant
pH	Prevalence	−0.015	0.952	No
	Shannon diversity	0.024	0.923	No
	Mean richness	0.096	0.695	No

Temperature	Prevalence	0.520	0.022	Yes ^∗^
	Shannon diversity	0.417	0.076	No
	Mean richness	0.509	0.026	Yes ^∗^

Humidity	Prevalence	−0.160	0.514	No
	Shannon diversity	0.084	0.733	No
	Mean richness	−0.231	0.342	No

Rainfall	Prevalence	0.607	0.006	Yes ^∗∗^
	Shannon diversity	0.276	0.253	No
	Mean richness	0.574	0.010	Yes ^∗^

*Note:* Spearman′s rank correlation, *n* = 19 sites.

Abbreviation: ns, not significant.

^∗^ *p* < 0.05,  ^∗∗^ *p* < 0.01.

A binomial GLM identified rainfall as the sole significant individual‐level predictor of infection (*β* = 0.740, OR = 2.096, 95% CI: 1.415–3.168, *p* < 0.001), indicating approximately twice the odds of infection at higher rainfall sites after controlling for host species (Table 7).

**Table 7:** Summary of the best‐fitting binomial GLM predicting infection status in urban anurans. Reference species: *Chalcorana raniceps*.

Predictor	*β*	SE	*z*	*p* value	OR	95% CI
Intercept	−0.233	0.530	−0.440	0.660	0.792	0.277–2.165
*D. melanostictus*	0.680	0.568	1.196	0.232	1.973	0.654–6.204
*F. limnocharis*	−1.125	0.733	−1.535	0.125	0.325	0.076–1.298
*K. pulchra*	1.176	0.701	1.677	0.094	3.241	0.851–13.419
Rainfall (scaled)	0.740	0.207	3.580	< 0.001 ^∗∗∗^	2.096	1.415–3.168

*Note:* Null deviance = 226.96 (df = 163); residual deviance = 194.63 (df = 159); AIC = 204.63.

Abbreviations: CI, confidence interval; OR, odds ratio; SE, standard error.

^∗∗∗^ *p* < 0.001.

NMDS ordination of the site‐by‐parasite matrix (19 sites × 10 taxa; Bray–Curtis) produced a two‐dimensional solution with near‐zero stress (stress = 0.0001). This likely reflects the low site‐to‐taxa ratio rather than a robust ecological signal; *envfit* results are, therefore, interpreted with caution and corroborated against Spearman′s correlation findings. Temperature (*r* ^2^ = 0.686, *p* = 0.001) and rainfall (*r* ^2^ = 0.479, *p* = 0.003) emerged as significant drivers of parasite community composition, whereas pH and humidity were not significant (both *p* = 0.070). Disturbance category did not significantly explain community variation (PERMANOVA: *F* = 1.094, *R* ^2^ = 0.120, *p* = 0.348) (Table 8).

**Table 8:** Results of envfit and PERMANOVA analyses examining environmental drivers of parasite community composition.

Variable	Statistic	*p* value	Significant
Envfit—environmental vectors			
Temperature	*r* ^2^ = 0.686	0.001	Yes ^∗∗∗^
Rainfall	*r* ^2^ = 0.479	0.003	Yes ^∗∗^
pH	*r* ^2^ = 0.292	0.070	No
Humidity	*r* ^2^ = 0.266	0.070	No
PERMANOVA			
Disturbance category	*F* = 1.094, *R* ^2^ = 0.120	0.348	No

*Note:* PERMANOVA: 999 permutations; Bray–Curtis dissimilarity; *n* = 19 sites.

Abbreviation: ns, not significant.

^∗∗^ *p* < 0.01,  ^∗∗∗^ *p* < 0.001.

Seven of the 10 recorded taxa have documented potential zoonotic concern: *Trypanosoma* spp., *Entamoeba* spp., *Macracanthorhynchus* spp., *Strongyloides* spp., *Ascaris* spp., *Trichuris* sp., and *Heterakis* spp. All seven were simultaneously harbored by *Duttaphrynus melanostictus*. The remaining three taxa (*Scheloribates* sp., *Hepatozoon* spp., and *Lankesterella* spp.) are not currently considered directly zoonotic (Table 9).

**Table 9:** Zoonotic status of parasite taxa recorded in urban anurans from southwestern Sarawak.

Parasite taxa	Parasite type	Potential zoonotic concern	Potential transmission to humans
*Trypanosoma* spp.	Blood protozoan	Yes	Through biting vectors (midges, bugs)
*Entamoeba* spp.	Gut protozoan	Yes	Through contaminated food or water
*Macracanthorhynchus* spp.	Gut helminth	Yes	Through ingestion of infected intermediate hosts
*Strongyloides* spp.	Gut helminth	Yes	Through direct skin contact with contaminated soil
*Ascaris* spp.	Gut helminth	Yes	Through ingestion of eggs in contaminated soil or water
*Trichuris* sp.	Gut helminth	Yes	Through ingestion of eggs in contaminated soil or water
*Scheloribates* sp.	Ectoparasite (mite)	No	Not reported in humans
*Hepatozoon* spp.	Blood protozoan	No	Not reported in humans
*Lankesterella* spp.	Blood protozoan	No	Not reported in humans

*Note:* Zoonotic status based on documented capacity to infect multiple vertebrate hosts including humans.

## 4. Discussion

In addressing the first objective, this study identified 10 parasite taxa across eight urban anuran species, representing ectoparasites, blood parasites, and gastrointestinal parasites, reflecting the diversity of transmission routes in urban environments. Eight anuran species were recorded across 19 urban localities, with community composition reflecting the dominance of generalist, urban‐tolerant species. Seven species are native and widely distributed across South and Southeast Asia [27, 28], and their persistence in disturbed habitats is consistent with broad ecological tolerances, including affinity for artificial aquatic habitats and dietary flexibility [29]. Four host species were represented by three or fewer individuals. Their low capture numbers reflect genuine rarity in heavily urbanized habitats rather than sampling inefficiency, as VES was applied consistently across all sites. These species are acknowledged as a limitation and excluded from statistical inference accordingly.

The overall infection prevalence is consistent with recent studies on tropical amphibians, where favorable climatic conditions sustain high parasite survival and transmission [9, 30]. *Kaloula pulchra* and *Duttaphrynus melanostictus* exhibited the highest infection rates, supporting the ecological amplification hypothesis, which proposes that urban‐adapted species accumulate more parasites due to frequent exposure to contaminated soil, a variety of invertebrate intermediate hosts, and higher densities of vectors that proliferate in disturbed landscapes [31, 32]. As an opportunistic terrestrial feeder commensal with humans, *Duttaphrynus melanostictus* is particularly exposed to STHs and beetle intermediate hosts of *Macracanthorhynchus* spp., whereas warty skin uniquely facilitates *Scheloribates* sp. attachment compared with smooth‐skinned species in this assemblage [29, 33–35]. In contrast, *Chalcorana raniceps* and *Fejervarya limnocharis* showed low prevalence, consistent with cleaner riparian microhabitat preferences and more effective antimicrobial skin defenses that limit parasite establishment [36].

Variation in individual counts across sites is acknowledged as an additional limitation that may influence site‐level Spearman correlations. However, this reflects the inherent patchiness of urban anuran assemblages rather than inconsistent effort. Given that opportunistic urban sampling will inevitably yield uneven representation, these results are best interpreted as a conservative baseline rather than a definitive account of community‐wide parasite structure.

With respect to the second objective, rainfall and temperature emerged as the primary climatic drivers of parasite infection, whereas humidity and pH showed no significant associations. By generating standing water and maintaining soil moisture, rainfall enhances the survival and density of moisture‐dependent vectors, such as biting midges (Corethrellidae) and hematophagous arthropods, and prolongs the viability of STH larvae in microhabitats frequented by *Duttaphrynus melanostictus* and *Kaloula pulchra* [37–39]. The consistency of this signal across both Spearman correlations and GLM analysis strengthens confidence in this finding despite the uneven site‐level sample size. However, it should be acknowledged that rainfall recorded at the time of sampling serves only as a proxy for the longer term moisture conditions influencing parasite transmission, as many parasite developmental stages and free‐living infective forms respond to accumulated environmental moisture rather than single rainfall events. Future studies should, therefore, consider using cumulative rainfall over 2–4 weeks preceding sampling to better capture the environmental conditions governing parasite survival and transmission.

Temperature structured parasite community composition most strongly across sites, acting as an environmental filter that determines which taxa can complete their life cycles at a given locality. Warmer sites support greater arthropod vector activity, particularly for blood parasites such as *Hepatozoon* spp. and *Trypanosoma* spp., while also potentially reducing the effectiveness of the ectotherm immune response [40–43]. Notably, this suggests that the urban heat island effect may indirectly amplify parasite persistence in heavily built‐up areas, even where direct disturbance effects are statistically weak, an underexplored but important mechanism linking land use change to parasite dynamics in tropical urban systems [38].

The absence of significant humidity and pH effects is ecologically consistent, as Sarawak′s persistently high ambient humidity reduces its power to discriminate between sites, making episodic rainfall a more ecologically meaningful predictor, whereas narrow pH ranges across sites provided insufficient variation for detection [9] Although infection prevalence increased progressively from low to high disturbance sites, this trend did not reach statistical significance, most likely due to the limited number of sites per disturbance category rather than genuine absence of effect. Urbanization has a well‐established association with elevated parasite burden through increased vector breeding habitat, immunosuppressive pollutants, and nutrient enrichment that support larval survival [1, 38]. The lack of significance is likely due to an uneven and limited number of sites per disturbance category, which reduces statistical power to detect real differences even when they exist. A larger and more balanced site design would be necessary to confirm or refute this relationship conclusively.

### 4.1. Zoonotic Relevance of Parasites Detected

With respect to the third objective, seven of the 10 recorded taxa were identified as having been documented as potential zoonotic concern species. However, parasite identification in the present study was based solely on morphological characteristics. Consequently, the detected taxa should be interpreted as belonging to genera that include zoonotic species rather than as confirmation of human infective species. Molecular characterization is required to verify species‐level identity and determine their actual zoonotic potential.

The simultaneous presence of seven potential zoonotic species in *Duttaphrynus melanostictus*, the most abundant species in this study, suggests a potential public health concern that warrants continued surveillance, particularly because this species commonly inhabits markets, drainage systems, and residential areas. STHs (*Strongyloides* spp., *Ascaris* spp., and *Trichuris* spp.) are established human pathogens transmissible through skin contact and ingestion of contaminated soil, disproportionately affecting children in communities adjacent to open drains [44, 45], precisely the environment where *Duttaphrynus melanostictus* and *Kaloula pulchra* are most abundant. *Entamoeba* spp. may include pathogenic strains transmissible via the fecal–oral route under inadequate sanitation conditions [46], whereas *Trypanosoma* spp. warrants monitoring given the proximity of competent vector insects to urban frog populations and the broad vertebrate host range of related species [47].

Parasite infection in urban anurans of southwestern Sarawak is driven by host identity, rainfall, and temperature rather than urbanization level alone. Among all species, *Duttaphrynus melanostictus* stands out as the most important host, carrying the widest range of parasites and living in closest contact with humans. Although uneven sample size limits some species‐level conclusions, the environmental patterns are consistent and reliable. These findings provide a much‐needed baseline for future monitoring and wildlife management in the region.

Several limitations should be acknowledged. First, four host species were represented by three or fewer individuals, limiting reliable prevalence estimation and preventing formal statistical comparisons. Second, the unequal number of sampling sites among urbanization categories reduced statistical power to detect subtle disturbance effects. Third, parasite identification relied solely on morphological characteristics, meaning species‐level identity and zoonotic potential require molecular conformation. Finally, rainfall measurements represented conditions during sampling rather than cumulative environmental exposure, which may more accurately reflect parasite development and transmission dynamics. Further studies incorporating larger sample sizes, balanced sampling designs, molecular barcoding, and cumulative climatic metrics would strengthen understanding of parasite ecology in tropical urban environments.

## 5. Conclusions

This study successfully characterized the community patterns of ecto‐ and endoparasite communities in urban anurans from southwestern Sarawak and identified host species identity, rainfall, and temperature as the primary determinants of infection prevalence and community structure. Ten parasite taxa were recorded across eight species, with *Duttaphrynus melanostictus* functioning as the principal reservoir host, harboring all taxa and the greatest coinfection burden. Although the urbanization disturbance category alone did not significantly explain parasite assemblage variation, this result is likely attributed to the limited site numbers per category rather than the absence of an urbanization effect. Seven taxa are of potential zoonotic concern, and their co‐occurrence in a human‐commensal species underscores the need for routine parasite surveillance as part of broader public health monitoring in Malaysian Borneo. The routine surveillance should include periodic monitoring of soil contamination in public parks, markets, and residential drainage systems where *Duttaphrynus melanostictus* commonly occurs, together with community education programs that reduce exposure to contaminated soil and standing water. Future studies should standardize sampling effort with a minimum of 10 individuals per species and a more balanced site distribution across disturbance categories to improve statistical power and reduce detection bias.

## Author Contributions

D.B.Q.A.A.R. wrote and revised the article. Z.H.A., A.H.A.I., and A.S.A.S. carried out the research and data collection. R.Z. provided expertise in anuran identification and confirmation of species. M.A. conceptualized the central research idea, provided the theoretical frameworks, designed the research, supervised research progress, and reviewed and revised the article.

## Funding

This study was funded by the Ministry of Higher Education Malaysia through the Fundamental Research Grant Scheme for Research Acculturation of Early Career Researchers, RACER/2019/SKK12/UNIMAS/1 and Universiti Malaysia Sarawak.

## Disclosure

Madinah Adrus approved the article submission.

## Ethics Statement

All procedures in this study have been permitted and approved by the Universiti Malaysia Sarawak Animal Ethics Committee, with permit number UNIMAS/AEC/R/F07/046, and approval and permission to conduct research on biological resources with Permit No. (137) JHS/NCCD/600‐7/2/Jld.2 by the Controller of Wildlife, Forest Department Sarawak.

## Consent

The authors have nothing to report.

## Conflicts of Interest

The authors declare no conflicts of interest.

## Data Availability

The findings are based on original data collected and analyzed using the methods described. The data are available upon request.
